# Application of Eco-Friendly Waterborne Polyurethane Composite Coating Incorporated with Nano Cellulose Crystalline and Silver Nano Particles on Wood Antibacterial Board

**DOI:** 10.3390/polym12020407

**Published:** 2020-02-11

**Authors:** Liangsong Cheng, Shaobo Ren, Xiaoning Lu

**Affiliations:** College of Materials Science and Engineering, Nanjing Forestry University, 159 Longpan Road, Xuanwu District, Nanjing 210037, China; chanson@126.com (L.C.); rshaobo@126.com (S.R.)

**Keywords:** nano cellulose crystalline, waterborne polyurethane, silver nano particles, antibacterial property, adhesion

## Abstract

To endow wood plate with antimicrobial properties, waterborne polyurethane (WPU) coatings incorporated with nano cellulose crystalline (NCC) and silver nanoparticles (AgNPs) were prepared. AgNPs were obtained by the chemical reactions of silver nitrate solution and sodium borohydride solution. The scribe testing results showed that the adhesion of the NCC-WPU composites was improved with the addition of NCC. The adhesion reached its peak when the amount of NCC added was 1%. Scanning electron microscopy (SEM) observation displayed that the NCC dispersed into the WPU without aggregation. NCC was well able to bind WPU and wood cell walls tightly together. Atomic force microscopy (AFM) and ultraviolet-visible (UV-vis) results revealed that WPU/NCC/AgNPs composites were homogeneous. This compatibility was also confirmed by transmission electron microscopy (TEM) analysis. The antibacterial property was improved too. When the adding amount of NCC was 0.5%, and the proportion of silver elements added was 5%, the antibacterial effect was at its best. As a comparison, the antibacterial effect of hybrid colloid without the addition of NCC was far less than that of including NCC. The WPU/NCC/AgNPs composite could be applied as an antibacterial coating in wood materials.

## 1. Introduction

Wood materials are widely used in architecture and decoration all over the world. We almost inevitably have many close contacts with it every day [1,2]. At the same time, various environmental pollution problems lead to the growth of various kinds of bacteria, which are all a threat for people’s life and health. Therefore, in the pursuit of the development of material and spiritual civilization, human health and safety should have the highest priority [3,4]. According to statistics, 90% of modern people’s time is spent in the indoor work and life, and 65% of that time is spent in the living room [5]. More studies have shown that many daily necessities that people frequently contact with during indoor activities have a variety of harmful pathogenic bacteria. The indoor furniture and finishing materials are the main transmission and survival media of microorganisms and bacteria that can affect human health in this atmosphere, and their common parts are also very easy to cause cross- infection [6,7]. In recent years, people’s health requirements for the indoor environment have risen from the initial safety and environmental protection of raw materials to the height of antibacterial and bactericidal furniture surface. The application of antibacterial technology on the floor and furniture has attracted more and more attention [8].

The board with surface antibacterial function not only retains all of the characteristics of traditional wood board, but also has the function of inhibiting and killing most harmful bacteria and inhibiting their growth and reproduction. In the future, antibacterial board will have a huge potential demand and broad development prospects [9].

Applying antibacterial coating on the surface of the material is the simplest and most efficient way to make them have antibacterial properties. Among wood coatings, waterborne polyurethane (WPU) is a kind of widely used one because of its environmental protection characteristics and easy construction, but the antimicrobial properties are very poor [10,11,12].

In order to improve the antimicrobial properties, many inorganic nanoparticles were introduced. Nano metal particles are a kind of important inorganic fillers, which have been successfully applied to the functionalization of polymer materials, bringing new functions to polymer materials [13,14,15]. However, the formation of aggregates will greatly reduce the applicability of inorganic nanoparticles. It is a great challenge to avoid agglomeration in the binding process of inorganic nanoparticles and host polymers.

Silver nanoparticles (AgNPs) are a widely recognized antimicrobial material because of their nontoxicity. The silver nanoparticle has numerous applications in many places such as food and other industrial products. Although there are many ways to prepare AgNPs, most of them are easier to form aggregates, which will greatly reduce the number of non-aggregates and the applicability of nanoparticles [16]. In order to minimize the aggregation of nanoparticles, the addition of a reducing agent in the presence of nanocellulose is the most effective method.

Under these conditions, nanocellulose crystalline (NCC) was introduced as a reagent because of its nanoscale effect to make the obtained AgNPs disperse better and blend better with WPU or other coatings. In addition, NCC can also be used as a reinforcing agent, which has been mentioned in many studies to improve the mechanical properties of nanocomposites. Mixed AgNPs and NCC have been used in many ways, even including wound dressing adsorbents in medicine [17]. Liu et al. [18] prepared a kind of material with carboxylated cellulose nanoparticles and AgNPs nanocomposites as dual functional nanofillers to improve the mechanical properties of nanocomposites and the antibacterial properties of WPU. The complexes have good antibacterial properties against gram-negative *Escherichia coli* (*E. coli*) and gram-positive *Staphylococcus aureus*. A mixture of nanocellulose and AgNPs nanocomposites not only exhibit good antibacterial function, but also improve mechanical properties, and thus they can be used in the field of active packaging films, coatings and adhesives [19].

At present, although there are many studies on the introduction of AgNPs and nanocellulose whiskers, most of them are aimed at the modification of the coatings themselves, and there is no specific application, especially in wood protection. For coatings used in different substrates, their performance and process will be very different. In this study, in order to make the coating meet the requirements of wood panels, we further optimized the process of mixed colloids. The AgNPs are achieved by the chemical reaction of silver nitrate and sodium borohydride in solution, and it can be achieved without particularly harsh conditions. Larch pine panels are selected as the samples. It was found that the modified WPU used in wood can not only effectively enhance the adhesion, but also makes itself and the wood board have excellent antimicrobial properties. This makes it possible for wood boards to have more choices for their antimicrobial properties. It is also very helpful for people’s health and the widespread use of wood boards.

## 2. Experimental

### 2.1. Materials

Waterborne polyurethane transparent wood primer (solid content of 36.8%) was supplied by the CARPOLY Chemical Group Co., Ltd. (Jiangmen, China), nanocellulose was acquired in Guilin Qihong Technology Co., Ltd. (Guilin, China), distilled water was homemade, silver nitrate (analysis pure) purchased in Tianjin Tianyi Industrial Technology Development Co., Ltd. (Tianjin, China), sodium borohydride (analysis pure) was provided by Dezhou fu kai Chemical Liability Co., Ltd. (Dezhou, China), anhydrous ethanol (analysis pure) was obtained from Nanjing Chemical Reagent Co., Ltd. (Nanjing, China), mixed phosphate PH buffer was offered by Shanghai Magnetic Chuangyi Instrument Co., Ltd. (Shanghai, China), nutritional agar was bought from Hangzhou Tian He Microbial Reagent Co., Ltd. (Hangzhou, China), while the *Escherichia coli* (CMCC (B) 44102) standard strain was procured from Xiamen Base Biotechnology Co., Ltd. (Xiamen, China). And Larch (*Larix gmelinii* (*Rupr.*) *Kuzen*) was purchased in the market, Northeast Xiaoxing’anling. All chemical agents were used without further purification.

### 2.2. Preparation of WPU/NCC Composites

The addition amount of NCC is 0.5% of the solid content of WPU, and the process is as follows. NCC (0.09 g) was added to the distilled water (5.0 g), and the NCC colloid was obtained by using the high speed shear mixer at the speed of 10,000 rpm for 10 min. After that, this colloid was dispersed in WPU (50.0 g) by stirring the mixture with an electric agitator at a speed of 200 rpm for 10 min. In addition, the ultrasonic homogenization processor was under the condition of 100–200 W, vibrating 4 s and interval for 4 s circularly, dispersed 20 min continuously, to get the WPU/NCC colloid.

### 2.3. Preparation of NCC/AgNPs and WPU/NCC/AgNPs Composites

The NCC/AgNPs and WPU/NCC/AgNPs composites which had different loadings of Ag nanoparticles as fillers were prepared as follows: Different volumes of silver nitrate solution (0.01 mol/L), V1 (0 mL), V2 (0.8 mL), V3 (2.6 mL), V4 (4.2 mL), V5 (6.0 mL), V6 (8.6 mL) were added separately to the NCC suspension and WPU/NCC composites separately and recorded. Afterwards, the sodium borohydride solution (0.01 mol/L) with the same volume was slowly dripped and added. At last, NCC/AgNPs and WPU/NCC/AgNPs composites with antibacterial modification was obtained by stirring with the electric mixer at the speed of 200 rpm for 10 min. Meanwhile, WPU/AgNPs composites were prepared by the same method as described above without NCC as the control group. The content of NCC was also 0.5%. 

### 2.4. Preparation of Antibacterial Plate

The larch specimens were machined into a size of 1000 × 100 × 4 mm, and by sanding the surface. According to the National Standards of the People’s Republic of China for the general preparation method of lacquer film, the wood surface was sprayed with the coating amount of 180 g/m^2^. The weight of the unpainted larch specimen was weighed first, and then the weight of the WPU/NCC/AgNPs composites sprayed evenly on the surface of the larch specimen was then recorded. The differential value between them must be controlled in a range from 17.90 g to 18.10 g. The specimen was then flattened into the drying kiln for curing and removed after 96 h. Each square specimen was machined into a round with a diameter of 25.0 mm and thickness 4.0 mm after curing. Test numbers were coded with the mass percentage of the silver element and NCC, named as 0%, 1%, 3%, 5%, 7%, 10% and no NCC, respectively.

## 3. Characterization

The surface of wood plate coated with modified WPU was characterized by a field-emission scanning electron microscope (FE-SEM, HITACHI 54800, JEOL, Tokyo, Japan) and the morphology of NCC/WPU/AgNPs was observed by transmission electron microscopy (TEM), using a JEOL 2100 microscope (JEOL, Tokyo, Japan) operating at 200 kV. The suspension was diluted to 0.1 wt % and deposited onto carbon-coated grids (300-mesh copper) before measurement. The surface of WPU coatings with and without modification was observed with the atomic force microscope (AFM, Shimadzu SPM9600, Kyoto, Japan) using a tapping mode of silicon probes under a 1 Hz scan rate. The optical image of WPU coatings with and without modification were measured by an ultraviolet-visible (UV-vis) spectrophotometer (Shimadzu 2550, Kyoto, Japan). In order to clearly demonstrate the transmittance of WPU coatings, the spectra of samples were continuously recorded within UV light in a range of 250–800 nm with a resolution of 0.08 nm. 

The adhesion of WPU coatings with and without modification was determined according to the National Standards of the People’s Republic of China GB/T 1720-2006, which technically corresponds to ASTM D3359-2009 standard tests. The diameter of the antimicrobial ring was measured by Vernier caliper in a bacterial environment after 48 h. Each specimen was calculated three times to get the average value.

## 4. Results and Discussion

### 4.1. Adhesion Properties of WPU/NCC Composites

**Figure 1:**
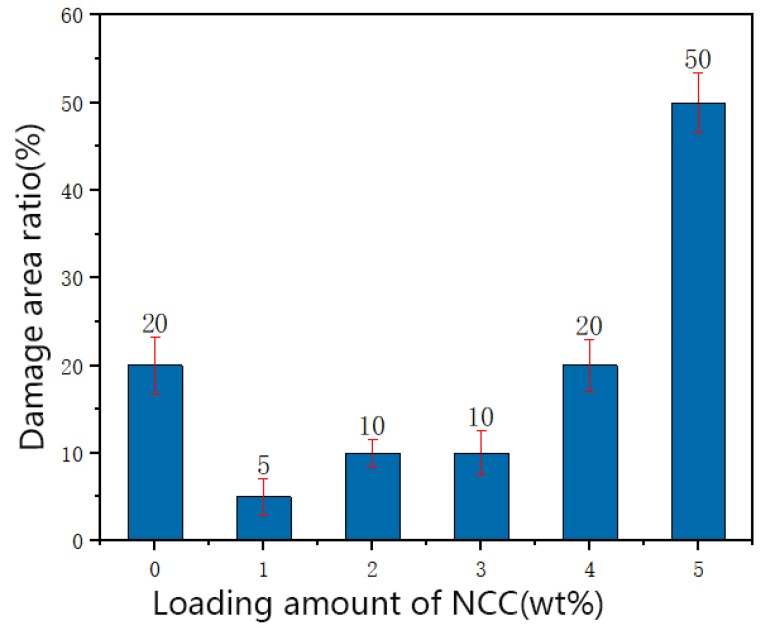
The results of scribe testing with different loadings of nanocellulose crystalline (NCC).

The behavior of the damage area ratio of WPU films and composite films reinforced with various compositions of NCC were investigated by scribe testing at room temperature. Results of scribe testing are presented in Figure 1 as a function of NCC content for the WPU matrix composite films. The damage area is reduced from 20% to 5% with the addition of NCC from 0% to 1%. This indicates that incorporating NCC into the WPU matrix results in strong interactions between the wood board and matrix, and thus improves the adhesion. Further addition of NCC increases the damage area ratio, and it even exceeds the damage area of neat WPU film when NCC is added from 4% to 5%. This result is consistent with the previous report. With the addition of NCC, the mechanical properties of WPU will increase, but when added to a certain extent, its mechanical properties will decrease with the addition of NCC [20,21,22]. In general, excessive NCC may interrupt the original interactions between soft and hard segments and be easy to aggregate [23,24].

### 4.2. Antibacterial Properties of WPU/NCC/AgNPs Composites

**Figure 2:**
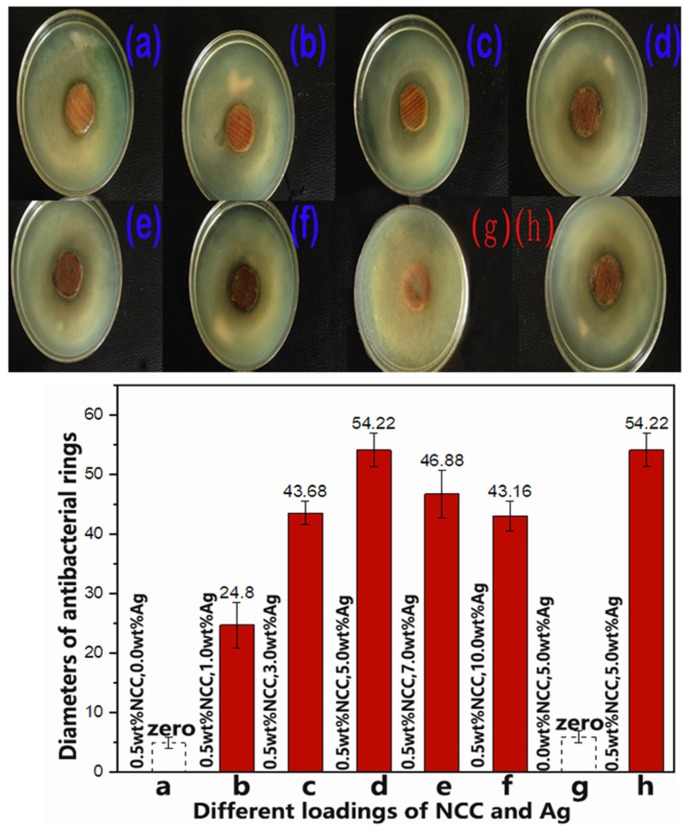
The results of antimicrobial experiment.

The diameter of the antibacterial ring is measured with a Vernier caliper, and the average data is obtained as shown in Figure 2. As can be seen from Figure 2, antimicrobial rings are formed around the specimens. However, due to the different amount of silver added in the lacquer film of each specimen, the diameter of the antibacterial ring around each specimen also varies. When the adding percentage of the Ag element rises from 0% to 5%, the diameter of the antibacterial ring increases gradually. However, when the silver element is added from 5% to 10%, the diameter decreases gradually. 

When the amount of silver added is the same (5%), the diameter of NCC added is much larger than that of without NCC. Thus, when the content of silver (5%) and NCC (0.5%) are added simultaneously, the diameter reaches its maximum and its antibacterial effect is the strongest. Therefore, NCC definitely plays a special role in it.

### 4.3. Morphology of WPU/NCC, and WPU/AgNPs/NCC

**Figure 3:**
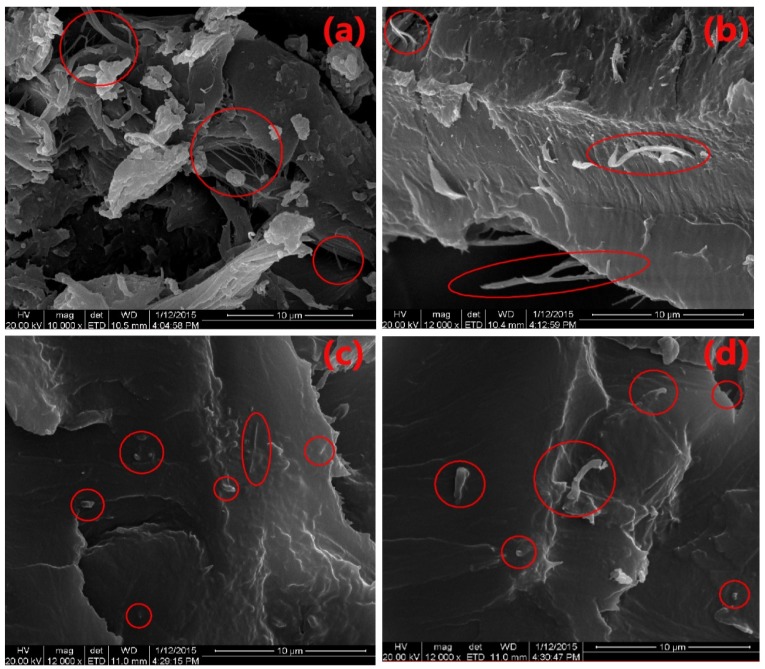
Scanning electron microscopy (SEM) images of nanocellulose crystalline (NCC) on the wood cell (**a**,**b**) and NCC-WPU (**c**,**d**).

Figure 3 illustrates the surface of NCC-WPU hybrid coatings. As can be seen from the figure, NCC does not appear as serious agglomeration but is evenly dispersed in the WPU. It can also be seen that some NCC is parallel to the surface of the specimen, the equivalent of lying flat in WPU, but there are many NCCs almost perpendicular to the surface of the specimen, that is, diagonally inserted in WPU. This is because when NCC is wrapped in WPU, the dispersion pattern is very random. But when one of the NCC has a hydrogen bond with a chemical group on the wall of a wood cell, NCC is not only affected by this hydrogen bond, but also wrapped in WPU. 

Under the pull of these two forces, many NCCs in WPU appear almost perpendicular to the surface of the plate state [21,25,26]. This also explains why the addition of NCC increases the adhesion of paint film.

**Figure 4:**
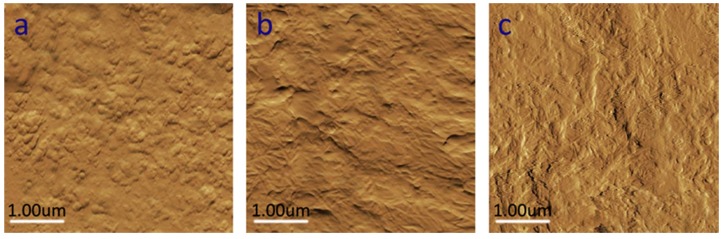
Atomic Force Microscopy (AFM)-micrographs of the surface of coatings: (**a**) WPU, (**b**) WPU/NCC, (**c**) WPU/NCC/AgNPs-5%.

The AFM image in Figure 4a shows that the surface of the pure WPU film is not very smooth, even rough, and no morphological changes due to the participation of NCC are observed. On the contrary, the rod shape of NCC can be easily identified in Figure 4b,c. This also shows that there is no NCC precipitation or flocculation in the evaporation process. It is observed that NCC is uniformly distributed in the WPU matrix, which indicates that the filler has good compatibility with the polymer matrix. To some extent, this can also explain why the adhesion of WPU/NCC hybrid coatings was improved. In Figure 4c, although the morphology of NCC is easy to identify, as the surface of WPU film itself is relatively rough, AgNPs cannot be clearly shown in the picture, the AgNPs in WPU/NCC/AgNPs composite membrane are not obvious and easy to find. Therefore, whether AgNPs can be dispersed in WPU/NCC/AgNPs needs further observation through other experiments.

### 4.4. Compatibility and Antibacterial Analysis

**Figure 5:**
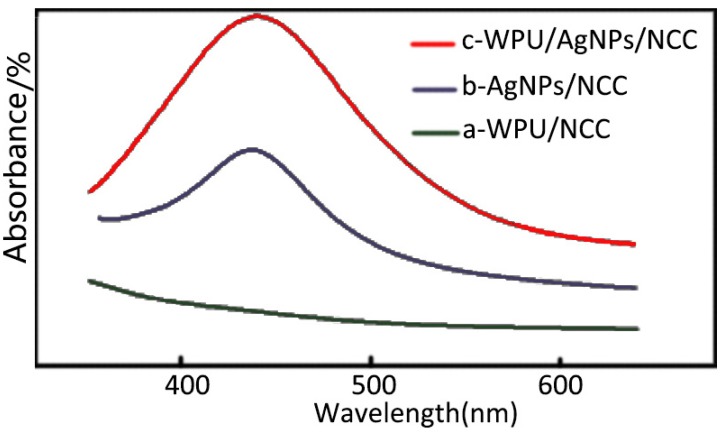
UV-vis spectra of (**a**) WPU/NCC, (**b**) NCC/AgNPs, and (**c**) WPU/NCC/AgNPs.

In order to further confirm the compatibility of AgNPs in WPU/NCC/AgNPs, a UV-Vis spectra experiment was carried out. The optical analysis of the composite film is shown in Figure 5. It can be seen that the WPU/NCC composite membrane has no absorption peak in the range of 300–600 nm (Figure 5a), while the WPU/NCC/AgNPs composite has an absorption peak at about 400 nm. The reason is that there is surface plasmon resonance absorption between 350–500 nm for AgNPs [18,27]. The absorption peaks of NCC/AgNPs and WPU/NCC/AgNPs composite films at about 400 nm belong to the surface plasmon resonance absorption of AgNPs. Through comparison, it can be found that there is no obvious displacement of the plasma resonance absorption peak of NCC/AgNPs and WPU/NCC/AgNPs composite films, which indicates that AgNPs are well dispersed in WPU, because the aggregation of AgNPs will lead to the migration of the surface plasma resonance absorption peak. Therefore, the compatibility of AgNPs in WPU composite can be confirmed through this experiment. Combined with Figure 4, it can be fully explained that both AgNPs and NCC can coexist well in WPU/NCC/AgNPs.

**Figure 6:**
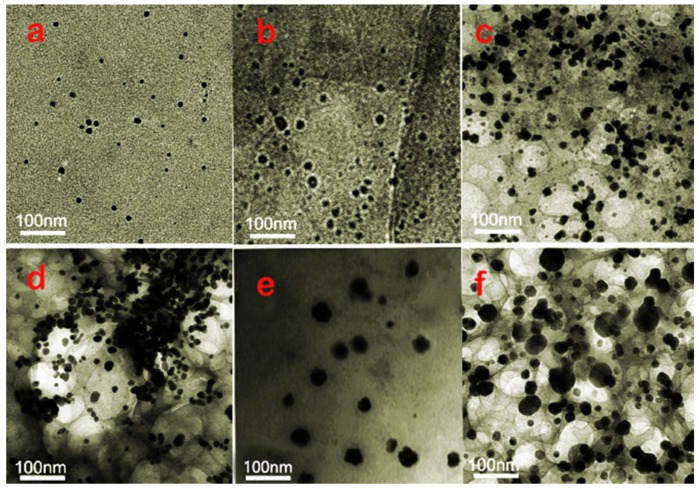
TEM images of WPU/NCC/AgNPs composites with 0.5% of NCC and different loadings of Ag: (**a**) 1%, (**b**) 3%, (**c**) 5%, (**d**) 5% without NCC, (**e**) 7%, (**f**) 10%.

Although it can be seen from the analysis of Figure 3, Figure 4 and Figure 5 that AgNPs, NCC and WPU can coexist well, it cannot be explained why the antibacterial effect of the different content of AgNPs is different. In order to further understand the antibacterial properties of AgNPs, TEM experiments were carried out. TEM images of WPU/NCC/AgNPs composites with 0.5% of NCC and different loadings of Ag was displayed in Figure 6. From previous studies, we know that NCC can be used as the coordination agent and carrier of Ag^+^ in solution because of the large amount of carboxyl and hydroxyl groups on the surface of NCC [18].

When NaBH_4_ was added with NCC and Ag^+^, the color of the solution changed to yellow rapidly (black when the concentration of Ag^0^ was high), indicating the formation of AgNPs. Both Ag^+^ and Ag^0^ are adsorbed by hydroxyl, which not only makes Ag^+^ adsorb on NCC, but also controls the growth of AgNPs and prevents the aggregation of AgNPs. Because NCC has a good dispersion in aqueous solution, the purified NCC/Ag complex also has a good dispersion in water. There is no flocculation and sedimentation in the dispersion after standing for two months. It can be seen from the diagram that the size of AgNPs prepared under different conditions ranges from several nm to dozens of nm, and the particle size of nanoparticles increases with the increase of Ag^+^ concentration during the preparation process.

It was clearly shown that the average size of AgNPs is less than 10 nm to 15 nm when the silver content goes from 1% (Figure 6a) to 5% (Figure 6b,c). Sizes of AgNPs increases to more than 50 nm when the silver content reaches 7% and 10% (Figure 6e,f). Average size of nanoparticles clearly increases with metal cation concentration during the synthesis. However, the silver particles gather seriously in the absence of NCC, resulting in many of its sizes exceeding 50 nm (Figure 6d). It can also be observed that AgNPs have good dispersion in the mixture, but NCC is not found in the figure because of the low contrast between NCC and the carbon support film. From the experimental results in Figure 2, we can see that with the increase of the content of AgNPs, the antibacterial performance is enhanced. At 5%, the antibacterial performance is the best. Subsequently, with the increase of AgNPs, the antibacterial properties decreased. 

The experimental results are in good agreement with the size and quantity of AgNPs in Figure 6. In fact, the antibacterial mechanism of AgNPs is to contact the outer membrane of bacteria through AgNPs, which further leads to bacterial death [28,29]. Meanwhile, AgNPs with a size less than 15 nm are well known to have efficient antibacterial activity because they are able to penetrate inside the bacteria and cause further damages, possibly by interacting with sulfur- and phosphorus-containing moieties of DNA. However, when the particle size is more than 15 nm, its bactericidal effect is significantly reduced, or there is even no bactericidal effect [18,19]. From the TEM diagram of AgNPs, it can be seen that when the Ag content is 1%, 3%, 5% (Figure 6a–c), respectively, the particle size of AgNPs is less than or close to 15 nm. As the content of silver increases fivefold from 1% to 5%, the particle size increases from a few nm to around 15 nm (Figure 6c). The number of AgNPs will also increase significantly. The antibacterial properties of the composite films increased with the increase of AgNPs. While when the content of Ag is 7% and 10%, the particle size of AgNPs obviously increases, many particles are much larger than 15 nm. With larger size particles, it is difficult to penetrate the bacterial outer membrane into the bacteria, so that the antibacterial performance is weakened. We can also find that when the content of AgNPs is 5% with the addition of 0.5%NCC, its size is less than 15 nm and its quantity is the most. Without NCC, although the amount of AgNPs is large, almost all of them gather together, and its scale is far more than 15 nm. This phenomenon illustrates that the antibacterial properties of WPU/NCC/AgNPs are ascribed to the size and quantity of AgNPs. It was explained that when the concentration of silver cation content is 5%, its antibacterial effect is strongest, and on the contrary, without NCC, its antibacterial performance is the worst, even close to zero.

## 5. Conclusions

In this paper, WPU/NCC/AgNPs nanocomposites were prepared, and the morphology, adhesion and antibacterial activity of WPU-based composites and neat WPU coated on the wood plate were investigated. AFM and UV-vis results indicate that NCC and AgNPs are dispersed homogeneously within the WPU matrix. It was learned from SEM that NCC can disperse in WPU evenly and bond with the wood cell. TEM results showed that with the increase of positive silver ions, the size AgNPs gradually increased. However, without NCC, AgNPs are very easy to aggregate when the content of silver is 5%. According to the results of paddle experiments, NCC had an important effect upon improving the adhesion of WPU. In this experiment, when the adding amount of NCC was 1 wt %, the adhesion was the best. More importantly, WPU/NCC/AgNPs hybrid coatings exhibit a strong antibacterial property against *E. coli*. It was also explained the application value of NCC, AgNPs or NCC/AgNPs nanocomposites as reinforced or antibacterial materials in wood WPU. By this way, the antibacterial property of wood board and paint film and the adhesion of paint film was improved. While providing a guarantee for human health, it also gives a new way for wood protection and utilization.
